# Feasibility of long-distance heart rate monitoring using transmittance photoplethysmographic imaging (PPGI)

**DOI:** 10.1038/srep14637

**Published:** 2015-10-06

**Authors:** Robert Amelard, Christian Scharfenberger, Farnoud Kazemzadeh, Kaylen J. Pfisterer, Bill S. Lin, David A. Clausi, Alexander Wong

**Affiliations:** 1 University of Waterloo, Department of Systems Design Engineering, Waterloo, N2L3G1, Canada; 2 University of Waterloo, Department of Kinesiology, Waterloo, N2L3G1, Canada; 3 University of Waterloo, Department of Mechanical and Mechatronics Engineering, Waterloo, N2L3G1, Canada

## Abstract

Photoplethysmography (PPG) devices are widely used for monitoring cardiovascular function. However, these devices require skin contact, which restricts their use to at-rest short-term monitoring. Photoplethysmographic imaging (PPGI) has been recently proposed as a non-contact monitoring alternative by measuring blood pulse signals across a spatial region of interest. Existing systems operate in reflectance mode, many of which are limited to short-distance monitoring and are prone to temporal changes in ambient illumination. This paper is the first study to investigate the feasibility of long-distance non-contact cardiovascular monitoring at the supermeter level using transmittance PPGI. For this purpose, a novel PPGI system was designed at the hardware and software level. Temporally coded illumination (TCI) is proposed for ambient correction, and a signal processing pipeline is proposed for PPGI signal extraction. Experimental results show that the processing steps yielded a substantially more pulsatile PPGI signal than the raw acquired signal, resulting in statistically significant increases in correlation to ground-truth PPG in both short- 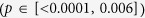 and long-distance 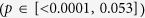 monitoring. The results support the hypothesis that long-distance heart rate monitoring is feasible using transmittance PPGI, allowing for new possibilities of monitoring cardiovascular function in a non-contact manner.

Photoplethysmography (PPG) is a non-invasive light-based method that has been used since the 1930s for monitoring cardiovascular activity^1^,^2^,^3^. These devices have been used to assess cardiovascular factors such as blood oxygen saturation, heart rate, autonomic function, and peripheral vascular disease^2^. Conventional contact PPG devices are fastened onto peripheral sites including the finger, ear, and toe^2^. Standard devices are comprised of either a single or multiple light-emitting diodes (LED) and a photodetector. These devices monitor cardiovascular activity by evaluating the change in light intensity resulting from temporal fluctuations in local blood volume. However, contact PPG devices are limited to single patient monitoring, resulting in increased cost per individual, and limiting the number of possible concurrent measurements. Furthermore, these devices produce single-point blood pulse measurements, which do not provide information about blood perfusion patterns which may be important for assessing tissue viability^4^. Finally, their contact nature makes them unsuitable for scenarios in which skin contact is problematic, such as neonatal monitoring, burn wound assessment, and sleep studies, as well as long-term continuous monitoring due to user comfort and movement artefacts^2^.

To address these issues, photoplethysmographic imaging (PPGI) systems have been proposed for non-contact heart rate monitoring. The goal of PPGI systems is to extract hemodynamic waveforms without intrusive tissue contact. Although existing designs differ, the primary components are analogous to contact PPG devices: a light source (LED) and a light detector (camera). Such systems rely either on active^5^,^6^,^7^,^8^,^9^,^10^ or ambient^11^,^12^,^13^,^14^,^15^ tissue illumination. Both active and ambient PPGI systems are sensitive to temporal changes in uncontrolled ambient illumination. Some studies mitigated this effect by using dark room settings for device validation^5^,^7^,^8^,^10^. Normalising ambient illumination changes using software has been proposed^15^, however this technique relies on a spectral estimation of the ambient illumination, which may fail in difficult lighting conditions.

A potential, yet largely uninvestigated, advantage of PPGI is the ability to perform long-distance monitoring (i.e., monitoring at the supermeter level). In contrast to existing short-distance PPGI monitoring, long-distance PPGI monitoring can enable whole-room monitoring and multi-individual assessment. This can be beneficial in settings such as assessing infants’ health conditions in a neonatal intensive care unit. Whole-room, multi-individual assessment is difficult or infeasible with contact PPG devices, since the devices are either are attached via a cable to a monitor, must store the data on the device, or must transmit the data wirelessly, resulting in the need for a specialised network infrastructure. Additionally, one contact device can only monitor one individual. This is also challenging in PPGI systems. Many existing PPGI systems operate in reflectance mode, where the camera and illumination source are positioned on the same side of the tissue under investigation. These reflectance PPGI systems have been limited in utility for long-distance monitoring due to two primary factors. First, the signal that is scattered back to the sensor is relatively low compared to the incident illumination. Light-tissue interaction can be summarized by the equation:


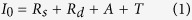


where *I*_0_ is the illumination incident on the skin, *R*_*s*_ and *R*_*d*_ are specular and diffuse reflectance, *A* is absorption and *T* is transmittance. Since the illumination source and sensor are positioned on the same side of the tissue in reflectance systems, the only component of the sensor irradiance that contains blood-related activities is *R*_*d*_ (some of which may have scattered prior to hemoglobin absorption), which has experienced scattering and absorption events due to the highly scattering nature of skin in visible and near infrared wavelengths^16^,^17^. Second, divergent illumination sources exhibit an intensity decrease proportional to the square of the distance, leading to reduced tissue irradiance at long distances. A second decrease happens from the tissue to the sensor, since the illumination source and sensor are positioned on the same side of the tissue in reflectance systems. These factors render long-distance monitoring a challenging problem in typical reflectance-based systems. These constraints may be alleviated using a transmittance PPGI system, where the LED is positioned behind a thin anatomical location (e.g., finger or toe), resulting in a lower number of scattering events and high hemoglobin absorption. To the best of the authors’ knowledge, this paper is the first to investigate long-distance transmittance PPGI monitoring.

This paper presents a pilot study to assess the feasibility of long-distance cardiovascular monitoring using transmittance PPGI. For this purpose, a novel non-contact transmittance PPGI system is proposed which is able to monitor cardiovascular activity remotely by correcting for ambient lighting fluctuations and extracting the subtle blood pulse signal using signal processing tools. This system comprises a 100 fps camera, a high-powered LED (655 nm), a microcontroller to synchronise frame captures and illumination, and image and signal processing software on a computer. Figure 1 shows a graphical representation of this system, and Figure 2 shows the processing steps. Temporally coded illumination (TCI) is introduced to remove ambient lighting artefacts at the acquisition level, thus correcting the data prior to further processing, similar to that introduced in our previous work^18^. The proposed PPGI system used for this study extends substantially beyond our previously reported work by incorporating a comprehensive set of signal processing steps, a larger testing sample size, and more rigorous statistical and qualitative analysis. The signal processing is particularly important for this study, as long-distance cardiovascular monitoring scenarios result in acquired signals with low signal-to-noise ratios (SNR).

## Results

### Experimental Setup

For this pilot study, five of the authors’ data (age 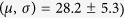 were collected using the proposed system. A 3:1 TCI code (alternating 3 frames with LED on, followed by 1 frame with LED off) was used with 100 fps frame rate and 8 ms exposure time. The aperture was manually adjusted to limit pixel saturation. The EasyPulse PPG device^19^ was used to collect the ground-truth PPG signal for validation. Approval for this study was obtained from the institution’s office of research ethics.

Two experiments were performed to assess the feasibility of long-distance monitoring. In Experiment 1, the camera and LED were separated by 20 cm, serving as short-distance base case validation. The participants were asked to position their fingers between the LED and camera so that their fingers covered the beam of the LED. In Experiment 2, the camera and LED were separated by 1.5 m (“long-distance”), and the participants were asked to position their fingers at approximately 10 cm from the LED. Figure 3 shows example images of both experiments before and after ambient correction. For each experiment, a 10 s window was chosen that yielded a clean ground-truth PPG signal for validation. The normalised power spectral density (PSD) was computed for spectral analysis to demonstrate each signal’s dominant frequency components. In an ideal signal, the fundamental heart rate should be the dominant frequency. Furthermore, to assess temporal signal fidelity, the Pearson’s linear correlation coefficient *ρ* was computed between PPGI and PPG signals. This metric is offset- and scale-invariant, suitable for the problem of comparing unit-less PPG signals:


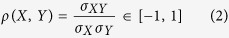


where *X*,*Y* are the finger PPG and PPGI signal, *σ*_*XY*_ is the covariance between the two signals, and *σ*_*X*_, *σ*_*Y*_ are the standard deviations of the same variables. This generates a value 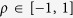, where 1 is perfect linear correlation, −1 is a perfect negative linear correlation, and 0 is uncorrelated. A perfectly reconstructed PPGI signal would yield *ρ* = 1, indicating that the PPGI signal is a linear scaling of the finger PPG signal.

The PPGI signals were statistically analysed using a novel physiologically-motivated *t*-test to quantify the degree to which the proposed system processing improved the signal. For each participant, each heart beat instance (i.e., diastole-to-diastole) was semi-automatically labeled using a custom gradient descent approach. *ρ* was computed for each such heart beat instance, yielding *n* correlation values. In order to assess an improvement in correlation after processing, a two-tailed paired-sample *t*-test (*α* = 0.05) was conducted using the correlation values for each heart beat instance, yielding a *p*-value indicating the probability of a zero-mean difference between signal correlation.

### Distance Parameters

It is important to study the effects of distance on the optical imaging parameters, primarily resolution and illumination intensity (i.e., irradiance). By calculating the area imaged by a single pixel, the spatial resolution of the imaging area can be determined. This can be computed using the optical magnification equation:


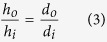


where *h*_*o*_, *h*_*i*_ are the object and image heights, respectively, and *d*_*o*_, *d*_*i*_ are the distances from the lens to the object and imaging sensor, respectively. According to the experimental setup, 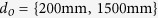 for short- and long-distance monitoring respectively, *d*_*i*_ = 6 mm (focal length of the lens), and *h*_*i*_ = 4.8 *μ*m pixel pitch. Then, the field of view of a single pixel is 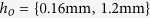 for short- and long-distance monitoring respectively. Thus, to measure a 40 mm × 40 mm area around the fingers, the resolution is 250 × 250 pixels for short-distance and 33 × 33 pixels for long-distance monitoring, yielding acceptable imaging resolution for spatial analysis.

Due to the inverse-square law of electromagnetic radiation, long-distance monitoring will exhibit greatly reduced irradiance relative to the source. Given two objects at distances *d*_1_ and *d*_2_ (*d*_2_ > *d*_1_) from an illumination source, noting the inverse-square law of intensity 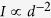, the change in illumination intensity can be computed as follows:


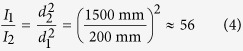


That is, the irradiance is reduced by a factor of 56 between long- and short-distance monitoring in the experimental setup.

### Experiment 1: Short-Distance

The goal of Experiment 1 was to validate the base case short-distance setup. Figure 4 shows the effect of processing the PPGI signal for three participants. The processed PPGI signal contained amplified pulsatility over the unprocessed (“raw”) PPGI. Pulsatile signals were extracted for all five participants (“P1” through “P5”), resulting in discernible systolic peaks of each blood pulse. Much of the noise was subdued upon processing. The high-frequency information not expected in the naturally smooth blood pulse waveforms was denoised, yielding smooth blood pulses. Furthermore, non-linear trends were corrected, yielding stable PPGI signals. These non-linear trends resulted in low correlation values between the raw PPGI and the ground-truth PPG (e.g., at 1.5 s for P3). The processed PPGI stabilised the signal, yielding larger correlation values at those time windows. The heart rate was discernible in both raw and processed PPGI as a peak frequency power coincident with the PPG peak. However, although all PPGI signals showed strong spectral similarity to the PPG signal in the PSD, the processed PPGI signal exhibited a stronger peak at the heart rate, thus subduing extraneous frequencies.

Table 1 summarises the statistical results for this experiment. The correlation values across whole-signal comparisons were substantially increased for each participant from unprocessed (*ρ* = 0.25 ± 0.21) to processed (*ρ* = 0.84 ± 0.08) due to recovered stability and pulsatility. This indicates a large correction across the entire signal, demonstrating an overall improvement in signal fidelity. Statistical analysis using the heart beat instance correlation values showed a statistically significant increase in correlation for each participant 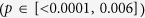. Thus, the resulting PPGI signal contained strong pulsatile components, enabling temporal inter-beat analysis.

### Experiment 2: Long-Distance

The goal of Experiment 2 was to validate long-distance PPGI measurement for long-distance monitoring. Figure 5 shows the effect of processing the PPGI signal for three participants. The raw PPGI signals were noisy, and pulsatility was not easily discernible in the signals of P1 and P3. This was emphasised by the lack of a distinct peak in the PSD. The processed PPGI signals yielded a higher degree of pulsatility, although the signals were noisier than the short-distance measurements (discussed later). This pulsatility was reflected in the PSDs, where there was a distinct peak coincident with the heart rate for each participant. However, in cases such as P2 and P3, distinct blood pulses were not easily observed, making beat-to-beat timing analysis difficult at long-distances. The correlation plots showed a general increase across each participant with processing, demonstrating the increased pulsatility. However, there were some areas that were not improved (e.g., P3 at 6–7 s), largely resulting from unsuppressed noise likely due to amplified movement artefacts (discussed later). This high-frequency noise did not have a major effect on the PSD within the physiologically valid heart rate range.

Table 2 summarises the statistical results for this experiment. As in Experiment 1, the correlation values for whole-signal results increased from unprocessed (*ρ* = 0.26 ± 0.14) to processed (*ρ* = 0.58 ± 0.17), indicating recovered pulsatility and stability for an overall increase in signal fidelity. Statistical analysis using the heart beat instance correlation values showed a statistically significant increase in correlation for each participant except P3 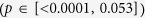. However, P3 exhibited substantial improvement upon processing (*p* = 0.053). This emphasises the increased beat-to-beat recovery of pulsatile components. Overall, correlation values were lower than for short-distance monitoring, but heart rate monitoring was achieved.

## Discussion

Strong results were obtained for short-distance measurement results, producing visually pulsatile signals and accurately extracting heart rate. Statistical significance was achieved 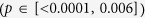, exemplifying the recovery of hemodynamic waveforms from a transmittance PPGI signal. The processed PPGI signal yielded smooth pulsatile components, making temporal beat-to-beat analysis feasible. For example, heart rate variability can be used as a measurement for autonomic nervous system activity^20^,^21^. The variability in correlation values across participants may be due to factors such as movement artefacts, and differences in scattering and absorptive media across tissues, such as melanin (skin tone) and adipose tissue^22^.

Long-distance monitoring yielded lower correlation values to ground-truth PPG than those for short-distance monitoring. Due to the diffuse nature of light traveling through highly scattering tissue^23^, the sensor irradiance is reduced, yielding weaker signals at greater distances. Furthermore, the area imaged by a single pixel increases with larger distances, thus small movements affected a larger number of pixels than in short-distance monitoring. This is apparent in the results for participant P3 (see Figure 5). The participant’s fingers were shaky, yielding high-frequency noise which the system was unable to fully remove. Automatic object tracking may alleviate this source of noise, During long-distance monitoring, statistical significance was achieved for heart beat instance correlation values in all but one participant (P3, *p* = 0.053), indicating the recovery of a PPGI signal.

Although temporal pulsatility was extracted in some but not all cases, the fundamental heart rate was identifiable across all participants. This indicates that beat-to-beat timing analysis becomes difficult, but heart rate is identifiable during long-distance PPGI monitoring. These results validate the feasibility of long-distance heart rate monitoring, but further work must be done to demonstrate beat-to-beat temporal analysis in long-distance monitoring. Such an imaging system could benefit settings in which the individual exhibits low motion, such as cardiovascular monitoring during neonatal monitoring or sleep studies. In both settings, minimizing the amount of tissue contact is preferable. Such an imaging system could provide physiologically relevant information in a non-contact manner. Additionally, the imaging system could monitor multiple individuals within the camera’s field of view by analysing each individual’s signal independently, thus decreasing the per-individual cost of the system.

The current system is best suited for situations in which there is minimal movement, such as in neonatal monitoring and sleep studies. In these cases, less expensive and simpler electrical and photoplethysmographic techniques may be problematic, as they require contact with the individual. In the case of neonatal monitoring, the infants’ skin may be too fragile for contact probes, and their size may not be suited for standard equipment. In the case of sleep studies, unnatural contact monitoring may hamper sleep quality, which can affect the data. In both of these cases, non-contact long-distance monitoring can provide essential physiological monitoring without inconveniencing the user.

The current PPGI system has three primary limitations. First, very bright ambient illumination may cause pixel saturation when using a fixed aperture setting. This can be mitigated with the use of optical filters, and automatic camera adjustment, such as exposure time and aperture. Second, physical obstructions may inhibit measurements. Long-distance transmittance PPGI requires an unobstructed view between the illuminated tissue and the camera. In many situations, this can be guaranteed by affixing the camera to the ceiling. However, there are settings in which completely unobstructed views are infeasible, especially in natural settings. In these cases, more advanced setups may be explored, such as the use of mirrors. Third, large movements may impact results. Automatic object tracking can be performed to track an area on an object in space over time, yielding spatially-invariant monitoring. These problems are grounds for future work.

## Methods

**Figure 1:**
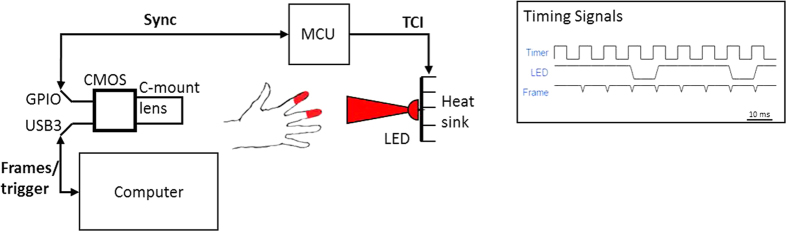
The proposed non-contact PPGI system comprises an LED (655 nm), a CMOS camera (100 fps), and a microcontroller (MCU) for synchronization and TCI implementation. The MCU timer dictates the TCI code, triggering the camera to acquire frames, which are transmitted and processed on the computer. All drawings were created by R.A.

**Figure 2:**
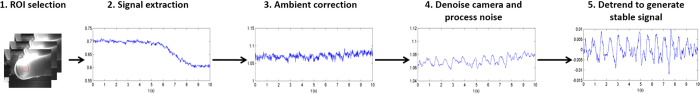
Overview of the processing steps for the proposed PPGI system. Upon acquiring frames, ambient correction using TCI is performed, which removes temporal changes in ambient illumination from the frames. This is followed by denoising to remove camera sensor noise and process noise from the light-tissue interaction, and smooth motion detrending. The resulting signal is an extracted stable PPGI signal.

To investigate the feasibility of long-distance PPGI monitoring, a non-contact PPGI system is proposed whose design integrates hardware (active illumination, a camera, and synchronisation electronics) and software (image and signal processing). Figure 1 depicts a graphical overview of the proposed PPGI system. A participant’s fingers were placed between a high-power LED (Philips LUXEON Rebel, 655 nm, 580 mW) and a camera (PointGrey Flea3, 100 fps) with a 6 mm lens. The camera frames and LED illumination patterns were synchronised by a microcontroller (MCU) and custom electronics. Temporally coded illumination (TCI) is proposed for controlling this illumination sequence. Figure 2 shows the stages of processing to yield the final PPGI signal. The following subsections provide detailed descriptions of the processing steps.

### Light Transport Model

A temporal extension of the Beer-Lambert law (BLL) was used. The BLL shows that the attenuation of light follows an exponentially decay as it passes through a homogeneous light-absorbing medium:


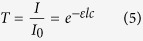


where *T* is transmittance, *I*_0_ is incident light, *I* is transmitted light, *ε* is the tabulated molar extinction coefficient for the light-absorbing medium (e.g., oxyhemoglobin), *l* is the photon path length, and *c* is the concentration of the medium. The temporal transmittance can be written as a function of time *t*:


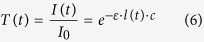


The path length for oxyhemoglobin is the dominant temporally changing parameter in heart beat analysis^3^. The standard measurement signal for PPG analysis is absorbance, which is related to transmittance as *A* = −log(*T*). Then, expressing this as temporal absorbance yields:


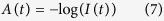


This model assumes constant ambient conditions. However, *I*(*t*) may be affected by temporal changes in ambient illumination, potentially corrupting the subtle blood pulse signal. Thus, in the next section, an ambient correction method is proposed.

### Temporally Coded Illumination (TCI)

A TCI process is proposed to remove the effect of ambient light changes in the data, thus ensuring that the reflectance signal is due solely to the controlled active illumination (e.g., LED). A dual-mode temporal code was defined by which frames contain irradiance due to either ambient illumination or a combination of ambient and active illumination. By coding the illumination synchronously with frame captures, ambient measurements can be performed according to the temporal code, and subtracted from the actively illuminated frames. For example, a temporal code of 3:1 defines a TCI sequence that measures one ambient frame after every three actively illuminated frames. Figure 1 shows a graphical example of 3:1 TCI.

Mathematically, given an intensity image *I*(*t*) at time *t*, according to the TCI sequence, *I*(*t*) can either be:


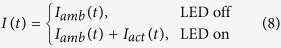


where *I*_*amb*_(*t*) and *I*_*act*_(*t*) are the irradiance due to ambient illumination and active illumination, respectively. Then, given a recently acquired ambient frame *I*_*amb*_(*t* − Δ*t*), by modeling light reflectance as a linear combination of ambient and active lighting, the ambient correction can be applied to the current frame:


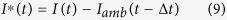


With a fast frame rate, Δ*t* becomes negligible, yielding illumination due solely to controlled active illumination:


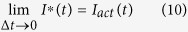


Since the camera operates at 100 fps, Δ*t* approximately satisfies this condition (Δ*t* = 10 ms). Substituting equation (9) into equation (7) yields the ambient-corrected absorbance measurement:


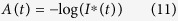


Once the ambient frame *I*_*amb*_(*t*) was acquired, the previously acquired active frame *I*(*t* − Δ*t*) was used in its place to ensure constant frame rate for frequency analysis.

**Figure 3:**
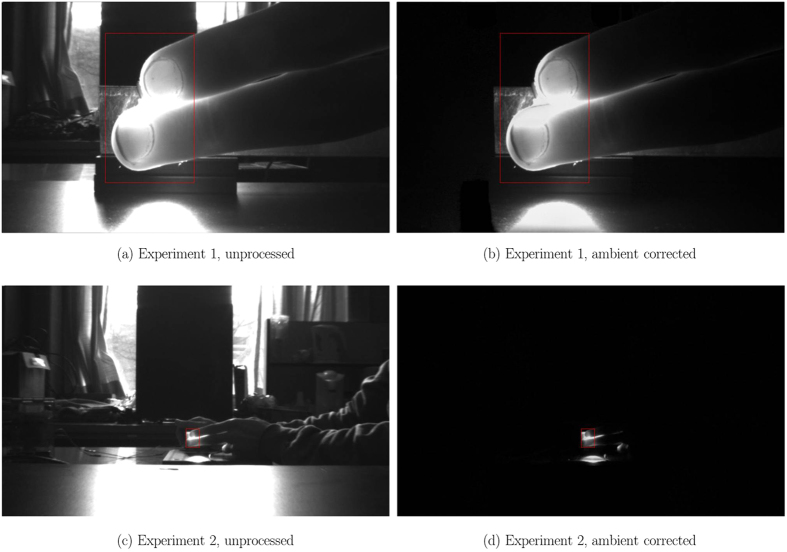
Example images for Experiment 1 (short-distance monitoring) and Experiment 2 (long-distance monitoring). The unprocessed frames (first column) contained uncontrolled ambient illumination (windows, overhead lights, etc.) as well as controlled active LED illumination near the fingers. Ambient correction using TCI (second column) removed the contribution of ambient illumination to the scene, yielding transmittance due solely to active LED illumination of which the spectral and power characteristics are known. See Video 1 and Video 2 for video results of ambient correction with varying illumination.

**Figure 4:**
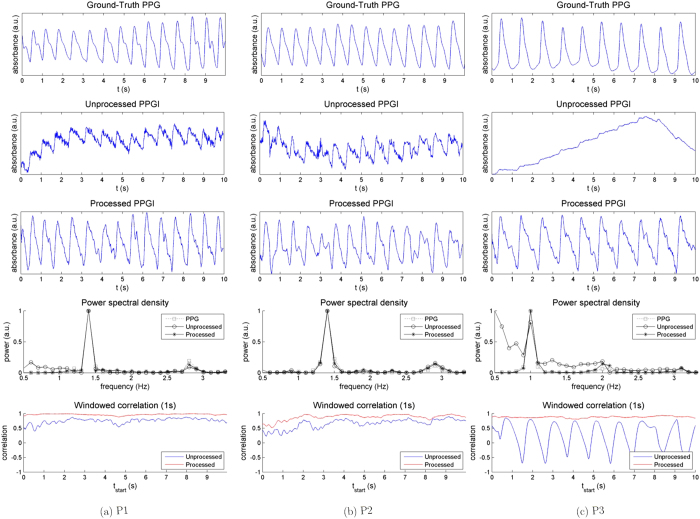
Summary of the results from Experiment 1 (short-distance measurement) across three participants (P1, P2, P3). The unprocessed acquired signal (row 2) was processed using ambient correction and signal processing, yielding a PPGI signal (row 3), which exhibited a higher correlation to the ground-truth contact PPG signal (row 1). For each participant, the PPGI power spectral density (row 4) closely matched the ground-truth PPG power spectral density, and the heart rate was easily distinguished by the maximum frequency power peak. The windowed correlation (row 5) of the processed PPGI signal with respect to the PPG signal (red) showed unanimous improvement over the unprocessed signal (blue), exemplifying the local temporal similarity across the entire signal.

**Figure 5:**
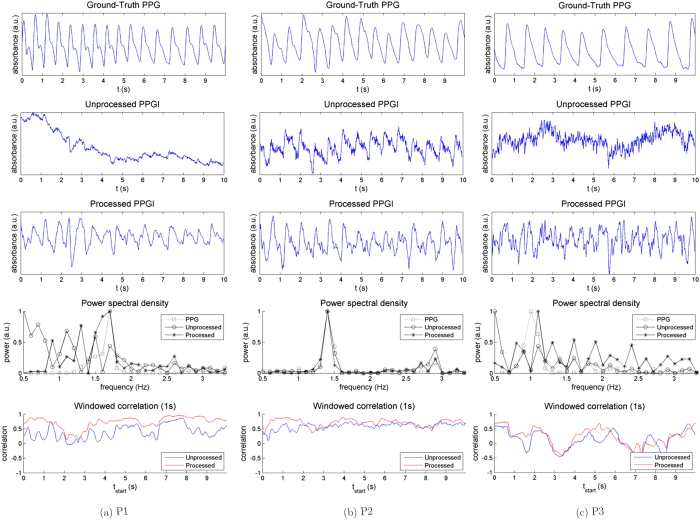
Summary of the results from Experiment 2 (long-distance measurement) across three participants (P1, P2, P3). The unprocessed acquired signal (row 2) was processed using ambient correction and signal processing, yielding a PPGI signal (row 3), which exhibited a higher correlation to the ground-truth contact PPG signal (row 1). The PPGI system was able to extract some pulsatile information from weakly pulsing (P1) and noisy (P2, P3) signals. As a result, the heart rate was determined through the maximum frequency power peak (row 4). The windowed correlation (row 5) of the processed PPGI signal with respect to the PPG signal (red) showed an overall improvement over the unprocessed signal (blue), exemplifying the local temporal similarity across the entire signal.

TCI was implemented at the hardware level, and requires control of an active light source. Thus an active illumination system was chosen. Camera and illumination synchronisation was performed using custom circuitry and microcontroller code. Figure 3 shows the results of ambient correction via TCI on a single frame. Video 1 and Video 2 demonstrate ambient correction across a series of frames with varying incident light.

### Signal Processing

Capturing data using the given light transport model and TCI yields an ambient-corrected PPGI signal. However, this signal may still be affected by various sources of noise. This section proposes a signal processing pipeline for extracting a stable pulsatile PPGI signal. First, a denoising step is proposed, which reduces the noise based on camera and process characteristics. Second, a detrending step is proposed, which removes trends in intensity due to external factors such as movement. This processing was performed using the average pixel intensity across a manually selected region of interest across the imaged fingers. See Figure 2 for a graphical depiction of this process.

### Denoising

The goal of signal denoising is to recover the smooth blood pulse waveform from a noisy signal. Kalman filtering^24^ was used since its model is consistent with PPGI denoising. First, its noise model assumes a combination of measurement noise and process noise. In this setup, measurement noise models camera read noise, and process noise models the stochastic process of light-tissue interaction. Second, Kalman filtering is a recursive algorithm that can operate in real-time, as denoising at time *t* depends only on the state and uncertainty matrix at time *t* − 1. Third, Kalman filtering models the system state based on Newton’s laws of physics, which describe smooth motion. This is well-suited for denoising PPGI signals, as blood pulse waveforms are inherently smooth in nature, with varying temporal acceleration.

Kalman filtering requires as inputs the system state and noise statistics. The system state was defined using Newton’s laws of motion using the raw camera intensity values:


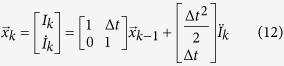


where, at time point *k*, *I*_*k*_ is signal amplitude, 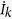 is the rate of change of the amplitude, and 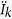 is acceleration of amplitude change. The measurement noise was assessed by imaging a dark field and quantifying the standard deviation of pixel intensities *σ*_*m*_ across the sensor. During the experiments, *σ*_*m*_ = 1.02 for 8 bit pixel values and 8 ms integration time. Denoising was performed on the raw camera signal *I*(*t*) rather than the computed absorbance signal *A*(*t*) since *σ*_*m*_ was learned using the untransformed raw camera intensity values.

Process noise is more difficult to model due to the complex and stochastic interaction of light with tissue. Thus, a grid search optimisation was performed to assess the optimal process noise value *σ*_*p*_ using 8 ms exposure time. Using a fixed measurement noise model and a training data set, a logarithmic grid search was performed over various values for *σ*_*p*_. The parameter value that yielded the highest correlation to the ground-truth PPG signal was chosen, resulting in *σ*_*p*_ = 1.84 × 10^−2^ for 8 bit pixels. This denoising process was then performed on the acquired signal using the standard recursive predict-update Kalman optimisation^24^. For each time point, the denoised intensity signal was extracted using an observation matrix:


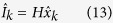


where *H* = [10]^*T*^, and 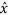 is the estimated true system state at time point *k*. Plugging this into equation (7) yields the denoised absorbance signal:


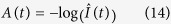


### Detrending

The denoising process assumes constant incident illumination, which is an invalid assumption during movement. The goal of this detrending step is to remove oscillations in the signal, yielding a PPGI signal with constant mean. It was assumed that movements are relatively smooth over time. A detrending algorithm was used on the denoised absorbance signal *A*(*t*) from equation (14), in which the model assumes a smoothness prior^25^. In particular, the observed signal *A*(*t*) is modeled as the linear combination of the “true” absorbance signal *A*_*true*_(*t*) and a temporal trend *A*_*trend*_(*t*).


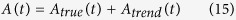


**Figure 6:**
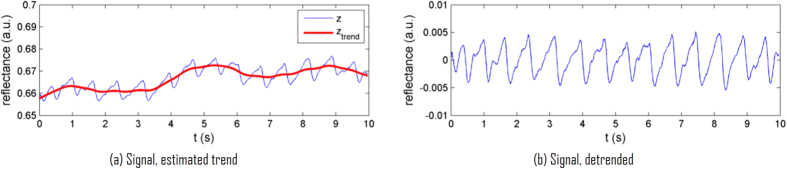
Example of the detrending procedure. A nonlinear trend was estimated in the original signal and subsequently removed, resulting in a signal with a stable mean. This process removed variability in illumination due to movement.

Given that *A*(*t*) is measured, *A*_*true*_(*t*) can be solved by estimating *A*_*trend*_(*t*) assuming a linear model, subtracting it from *A*(*t*), and solving this using regularised least squares, which provides the following estimate of the true detrended signal:


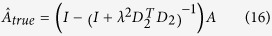


where *I* is the identity matrix, *λ* is a relative weighting term, and *D*_2_ is the discrete approximation matrix of the second derivative. Figure 6 shows the recovery of a stable signal from a signal corrupted by movement using this detrending method. 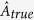 is the final processed PPGI signal.

## Additional Information

**How to cite this article**: Amelard, R. *et al.* Feasibility of long-distance heart rate monitoring using transmittance photoplethysmographic imaging (PPGI). *Sci. Rep.* **5**, 14637; doi: 10.1038/srep14637 (2015).

## Supplementary Material

Supplementary Video 1

Supplementary Video 2

## Figures and Tables

**Table 1:** Experiment 1 statistical results for short-distance measurements.

	P1	P2	P3	P4	P5
Unprocessed, whole	0.45	0.49	−0.02	0.19	0.16
Processed, whole	0.94	0.85	0.86	0.71	0.85
Unprocessed, blocked*	0.75 ± 0.09	0.66 ± 0.16	0.23 ± 0.44	0.53 ± 0.27	0.56 ± 0.15
Processed, blocked*	0.96 ± 0.02	0.88 ± 0.10	0.88 ± 0.03	0.77 ± 0.13	0.87 ± 0.07
*p*-value	<0.0001(*n* = 13)	<0.0001(*n* = 14)	<0.0001(*n* = 9)	0.006(*n* = 10)	<0.0001(*n* = 13)

Pearson’s linear correlation coefficient (*ρ*) was computed between the PPGI and PPG signals for the whole 10 s signal for global accuracy, and for each heart beat instance (i.e., diastole-to-diastole) for local accuracy. A *p*-value was computed using a two-tailed paired-sample *t*-test to test whether signal processing improves the PPGI correlation (*H*_0_: *μ*_*post*_ − *μ*_*pre*_ = 0). At the whole-signal level, the proposed system processing yielded improved accuracy and high correlation for each participant. At the local level, statistically significant results (*α* = 0.05) were achieved for each participant, indicating significant improvement with processing.

^*^mean ± *s*.*d*.

**Table 2:** Experiment 2 statistical results for long-distance measurements.

	P1	P2	P3	P4	P5
Unprocessed, whole	0.19	0.49	0.16	0.29	0.19
Process, whole	0.62	0.65	0.32	0.76	0.53
Unprocessed, blocked*	0.39 ± 0.23	0.59 ± 0.11	0.11 ± 0.32	0.49 ± 0.18	0.24 ± 0.23
Processed, blocked*	0.73 ± 0.20	0.71 ± 0.13	0.23 ± 0.30	0.79 ± 0.14	0.47 ± 0.28
*p*-value	< 0.0001(*n* = 15)	0.04(*n* = 13)	0.053(*n* = 9)	0.0001(*n* = 10)	0.0005(*n* = 11)

Pearson’s linear correlation coefficient (*ρ*) was computed between the PPGI and PPG signals for the whole 10 s signal for global accuracy, and for each heart beat instance (i.e., diastole-to-diastole) for local accuracy. A *p*-value was computed using a two-tailed paired-sample *t*-test to test whether signal processing improves the PPGI correlation (*H*_0_: *μ*_*post*_ − *μ*_*pre*_ = 0). At the whole-signal level, the proposed system processing yielded improved accuracy over the unprocessed signal for each participant. At the local level, statistically significant results (*α* = 0.05) were achieved for each participant except P3 (*p* = 0.053), indicating substantial improvement with processing.

^*^mean ± *s*.*d*.
